# Cycle-based high-intensity sprint exercise elicits acute cognitive dysfunction in psychomotor and memory task performance

**DOI:** 10.3389/fcogn.2024.1419734

**Published:** 2024-07-23

**Authors:** Trevor J. Dufner, Jessica M. Moon, Adam J. Wells

**Affiliations:** Exercise Physiology Intervention and Collaboration Laboratory, Institute of Exercise Physiology and Rehabilitation Science, University of Central Florida, Orlando, FL, United States

**Keywords:** cognition, cognitive dysfunction, high-intensity sprint exercise, ANAM, Dynavision, memory, attention

## Abstract

**Purpose:**

To examine the impact of an acute high-intensity sprint exercise protocol (HISEP) for eliciting post-exercise cognitive dysfunction in psychomotor, attentional, executive, and memory tasks.

**Methods:**

Twenty-four recreationally active adults (22 ± 4 yrs, 169.39 ± 10.07 cm, 75.80 ± 14.73 kg, 27.03 ± 9.55 BF%) performed a HISEP on a cycle ergometer. Average psychomotor reaction time (avgRT; Dynavision D2 Mode A & Mode B), mood (Profile of Mood States Questionnaire; POMS), and cognition (Automated Neuropsychological Assessment Metrics; ANAM) were assessed pre- (PRE), post- (POST) and 60-min post (60POST) HISEP. One-way repeated measures ANOVAs were used to assess changes across time.

**Results:**

Fatigue (main effect: *p* < 0.001, $\eta_{\text{p}}^{2}$ = 0.309) was significantly higher at POST compared to PRE (*p* = 0.007). Tension (main effect: *p* = 0.021, $\eta_{\text{p}}^{2}$ = 0.154) was significantly lower at 60POST compared to PRE (*p* = 0.029). Mode A avgRT (main effect: *p* = 0.022, $\eta_{\text{p}}^{2}$ = 0.153) was significantly slower at POST compared to PRE (*p* = 0.026). Throughput (TP) scores for ANAM code substitution-delayed (CSD) task (main effect: *p* < 0.001, $\eta_{\text{p}}^{2}$ = 0.284) and matching to sample (M2S) tasks (main effect: *p* = 0.014, $\eta_{\text{p}}^{2}$ = 0.169) were significantly lower at POST compared to PRE (*p* = 0.001 and *p* = 0.025, respectively), while mathematical processing (main effect: *p* = 0.002, $\eta_{\text{p}}^{2}$ = 0.232) was significantly higher at 60POST compared to both PRE (*p* = 0.019) and POST (*p* = 0.005). No other significant changes in cognitive task performance were observed (*p*'s > 0.05).

**Conclusions:**

The HISEP is a feasible and time-effective fatiguing exercise stimulus capable of eliciting acute cognitive dysfunction in psychomotor and memory task performance. NCT05100589.

## Introduction

Cognition is typically conceptualized as a comprehensive process consisting of multiple domains and subdomains working together synergistically to achieve optimal thinking, perception, and reasoning (Harvey, 2019). Acute high-intensity exercise has been shown to alter cognition and elicit physiological changes in the brain by perturbing cerebral blood flow, hormone concentrations, and neuronal metabolic requirements (Dietrich and Audiffren, 2011; Seifert and Secher, 2011; Sudo et al., 2022). As discussed in several reviews and meta-analyses (Lambourne and Tomporowski, 2010; Chang et al., 2012; McMorris and Hale, 2012; McMorris et al., 2015; Browne et al., 2017; Sudo et al., 2022), a variety of moderators such as cognitive task type, fitness level of the sample population, exercise intensity, and the exercise modality itself complicate the relationship between acute exercise and cognition, as well as the magnitude of transient changes in cognitive function that an exercise bout elicits. For example, acute moderate intensity aerobic exercise is typically observed to have a positive effect on most cognitive tasks (Lambourne and Tomporowski, 2010; Chang et al., 2012; McMorris and Hale, 2012). Alternatively, acute bouts of high-intensity to maximal or exhaustive aerobic exercise are less well characterized (Sudo et al., 2022), although the existing data generally indicates facilitation of memory tasks but diminished psychomotor function (Sudo et al., 2022). The duration of high-intensity protocols in the literature ranges from 8 to 60 min and while most published protocols end at volitional exhaustion, the reported intensities are highly variable, likely influencing the subsequent cognitive outcomes.

Many investigations have cited the inverted-U theory to explain post-exercise cognitive perturbations (McMorris and Hale, 2012; Moreau and Chou, 2019; Razon et al., 2019). In this model, the level of arousal elicited by moderate intensity exercise generally facilitates cognition, whereas further increases in arousal in response to increasing exercise intensity would have deleterious effects on cognitive function (Moreau and Chou, 2019). The findings of recent investigations, however, do not fully support this theory (Browne et al., 2017; Razon et al., 2019; Sudo et al., 2022). Alternatively, Dietrich and Audiffren (2011) posit that changes in cognition occur as a result of metabolic competition wherein the greater motor and sensory demands of high-intensity exercise take priority over prefrontal higher order cognitive processes (Dietrich and Audiffren, 2011), otherwise known as the hypofrontality hypothesis. In this model, tasks that are more automatic in nature are generally unaffected or even augmented by exercise, while tasks requiring higher prefrontal and executive functioning are more likely to be impaired by acute exercise (Dietrich and Audiffren, 2011). This model is more consistent with the findings of previous examinations showing null or positive effects of high-intensity exercise on basic tasks (simple reaction time and attention) (Mekari et al., 2015; Sudo et al., 2022) as well as detrimental effects on both psychomotor (Sudo et al., 2022) and higher order executive tasks (Labelle et al., 2013), especially during high-intensity exercise (Labelle et al., 2013; Dufner et al., 2023). However, neither of these models fully explain the equivocal findings of many previous investigations examining cognition immediately following exercise (Lambourne and Tomporowski, 2010; Chang et al., 2012; McMorris and Hale, 2012; McMorris et al., 2015; Browne et al., 2017; Sudo et al., 2022). Moreover, in a recent review of the literature, Sudo et al. (2022) were unable to find any definitive associations between acute high-intensity exercise and cognitive task performance following exercise, with the exception of memory tasks, which were generally facilitated. As such, the complex interaction between exercise and cognitive task performance, particularly following high-intensity exercise are yet to be fully elucidated (Lambourne and Tomporowski, 2010; Browne et al., 2017; Sudo et al., 2022).

The high-intensity sprint exercise protocol (HISEP) is a short duration supramaximal exercise protocol based on the 3-min all-out test typically used to estimate critical power, which requires participants to maintain a maximal effort for the entirety of the test (Vanhatalo et al., 2007; Moon et al., 2023). The HISEP has been reported to elicit reductions in anaerobic power output of up to 69% in recreationally fit individuals (Vanhatalo et al., 2007) making it an ideal candidate as a time efficient high-intensity exercise stressor. The HISEP is also a relatively easy protocol to administer, requires minimal familiarization, and only requires effort for 3 min. Mekari and colleagues (Labelle et al., 2013) have previously demonstrated that increasing the intensity of an exercise bout compounds the potency of its effects on cognition. In their investigation, 19 young adults underwent a continuous graded exercise test on a cycle ergometer at intensities of 40%, 60%, and 85% peak power output (PPO) during which their executive function was measured using a computerized Stroop test. Reaction time and accuracy were significantly influenced by exercise intensity with significantly larger deficits in performance being noted in the 85% PPO group compared to the 40% PPO group. Correspondingly, the HISEP may be capable of eliciting transient perturbations to the cerebral environment that could ultimately lead to acute cognitive dysfunction (Dietrich and Audiffren, 2011). Considering this, time conscious researchers may see utility in a short duration maximal exercise stressor such as the HISEP when seeking to elicit cerebral perturbations within a healthy recreationally fit population for the purpose of examining the impact of nutritional or supplemental interventions designed to attenuate cognitive dysfunction.

Currently, the complex relationship between high-intensity exercise and cognitive task performance/cognitive domains is somewhat convoluted due to ambiguity regarding the definitional descriptions of cognitive tasks and the domains they belong to, which has led to inconsistencies in the classification of cognitive tasks. Accordingly, previous studies examining the relationship between high-intensity exercise and cognition have yielded contrasting interpretations of findings. Furthermore, considering many researchers have posited that the type of cognitive task in addition to several other methodological factors heavily moderate the magnitude and direction of perturbance to a cognitive function assessment following acute high intensity exercise (Lambourne and Tomporowski, 2010; Chang et al., 2012; McMorris and Hale, 2012; McMorris et al., 2015; Browne et al., 2017; Sudo et al., 2022), future investigations would benefit from a methodological study that examines the effects of a relatively simple and highly repeatable high-intensity exercise stressor on specific cognitive task performance rather than making generalizations of these assessments to their respective hypothesized domains. Moreover, considering time elapsed between the exercise stressor and cognitive assessments is of similar importance to this relationship (Sudo et al., 2022), an investigation that provides a clear and detailed cognitive task timeline as well as an additional post-exercise cognitive assessment time point would provide greater context regarding the transiency of cognitive perturbations following exercise.

Previously, we examined the influence of adenosine 5′-triphosphate supplementation on reaction time (RT) and cognition following a three-minute HISEP in a smaller cohort of 20 subjects (Moon et al., 2023). Although we observed significantly reduced Mode A performance post-exercise that continued through a 60-min follow up time point in the placebo group, the reduced performance in many of the attentional and executive function tasks reported were collapsed across both supplement and placebo treatments. Thus, reported outcomes were not entirely independent of any potential treatment effects that just may not have reached significance. Further, no independent statistical analysis was performed in the placebo group for any cognitive variable in the Automated Neuropsychological Assessment Metrics (ANAM) test battery. As such, an independent analysis of the placebo group with a larger n size may provide a more objective assessment of the effects of the HISEP on cognition. Therefore, the purpose of this investigation was to examine the impact of the HISEP in eliciting post-exercise cognitive performance in a variety of sequential cognitive tasks. We hypothesized that the likely cerebral perturbations evoked by the HISEP would be substantial enough to elicit significant but transient post-exercise changes in attentional, psychomotor, memory, and executive function tasks.

## Materials and methods

### Experimental design

**Figure 1:**
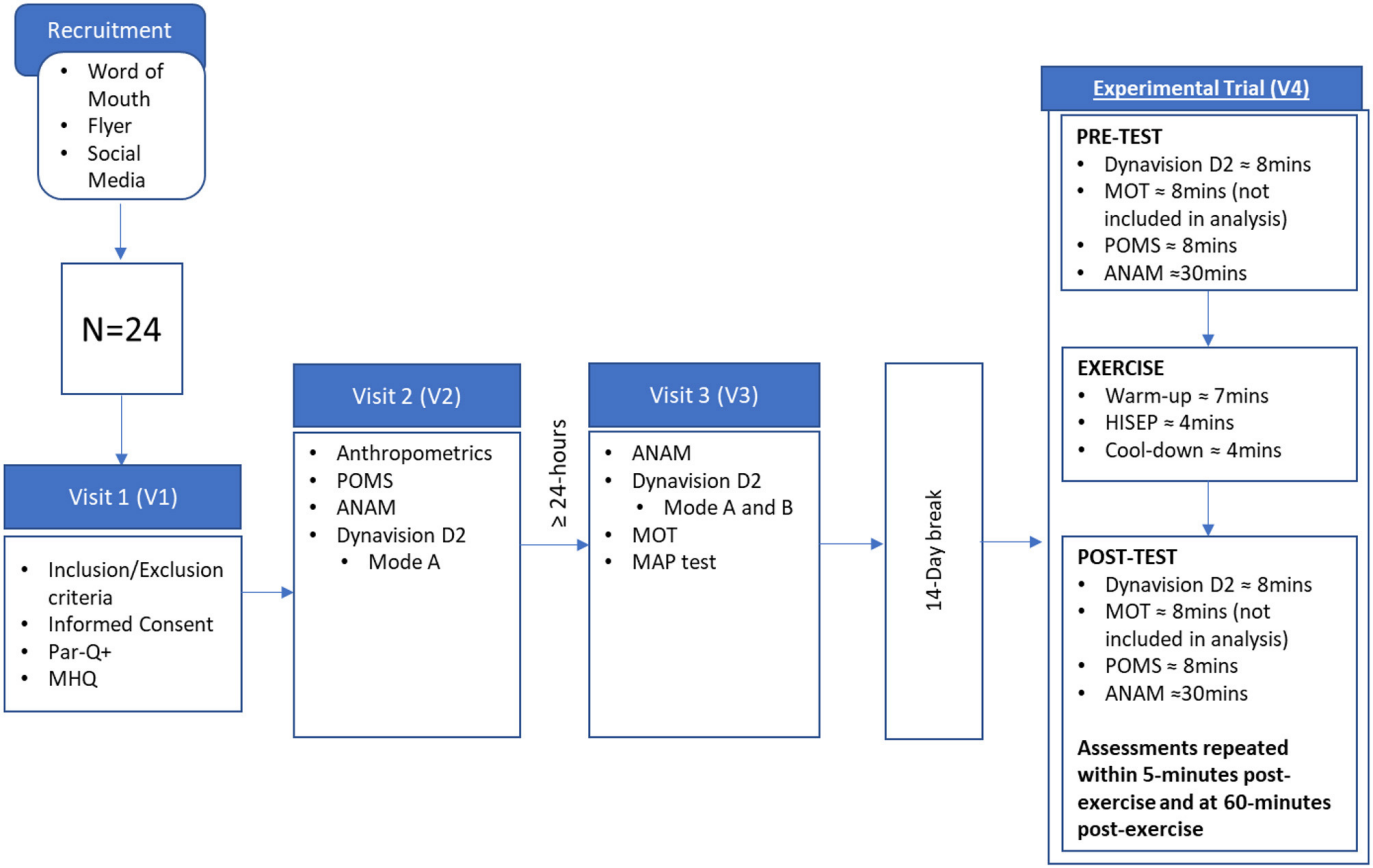
Research design overview. MHQ, medical health questionnaire; POMS, profile of mood states; ANAM, automated neuropsychological assessment metrics; MOT, multiple object tracking; MAP, maximal aerobic power.

This study followed a within group repeated measures design and was approved by the university's Institutional Review Board (STUDY00003272). Participants completed a total of 4 visits to the study site. Visit 1 (V1) consisted of an informed consent and screening to determine eligibility to participate in the study. Following V1, eligible participants were scheduled for two familiarization visits (V2 and V3) and instructed to abstain from caffeine ingestion for 24-h prior to all subsequent visits (V2-V4). During the first familiarization visit (V2), participants underwent anthropometric assessments of height, weight, and body composition via bioelectrical impedance analysis and were familiarized with the Profile of Mood States questionnaire (POMS), the ANAM assessments, and the Dynavision D2 (D2) Mode A psychomotor reaction time assessment. At least 24 h later, participants returned for the second familiarization visit (V3) to complete additional familiarization with the ANAM and D2 Mode A assessment and an initial familiarization with the D2 Mode B psychomotor reaction time assessment. Additionally, a maximal aerobic power (MAP) test was performed where power at the gas exchange threshold and peak power values were used to calculate the resistance for their HISEP to be completed during visit 4 (V4). In accordance with the requirements of a larger experimental design (Vanhatalo et al., 2007) from which this investigation originated, participants then returned to the lab 14 days later for V4 to complete their HISEP along with measures of mood (POMS), psychomotor reaction time (Dynavision Mode A and Mode B), and cognitive function (ANAM), which were assessed pre- (PRE), post- (POST), and 60 min post-HISEP (60POST). As part of this study, participants were familiarized with a multiple object tracking (MOT) assessment (attention domain) during V3 and completed a MOT assessment between POMS and D2 assessments during V4. The MOT assessment was approximately 8 min in duration and has been described previously (Renziehausen et al., 2022). MOT data is not included here as subsequent research from our lab indicates more extensive familiarization than was performed is required to eliminate learning/training effects that are evident with repeated measures designs (Moon et al., 2024). An outline of the study procedures is presented in Figure 1.

### Participants

An a priori power analysis using power analysis software (G^*^Power 3.1.9.4, HHU, Dusseldorf, Germany) revealed that for a paired-sample *t*-test, power of 0.95, *p*-value of 0.05, and an effect size (dz) of 0.963 derived from changes in Go/No Go accuracy from pre- to post- acute high-intensity exercise under normoxic conditions reported by Sun et al. (2019) a sample size of 14 would be required. A total of 24 healthy recreationally active adults (13 women & 11 men) between the ages of 18 and 40 (22 ± 4 yrs, 169.39 ± 10.07 cm, 75.80 ± 14.73 kg, 27.03 ± 9.55 BF%) successfully completed the study protocol. Prior to completing the study, participants provided written informed consent and were permitted to participate if they were free from all pulmonary, cardiovascular, autoimmune, musculoskeletal, gastrointestinal, or other diseases and disorders. To meet inclusion criteria and be considered recreationally active, participants had to report performing at least 150 min of combined physical activity each week according to the American College of Sports Medicine standard for recreationally active individuals. Readiness to perform physical activity was determined through completion of the PAR-Q+ assessment. Participants were required to abstain from supplementing with creatine and beta-alanine for a minimum of 4 weeks prior to beginning research protocols. The study sample of the current investigation includes placebo data from a larger intervention study (*n* = 20) from which two papers have been previously published (Dufner et al., 2023; Moon et al., 2023). The current investigation includes four additional participants not included in previously published data.

### Procedures

#### Anthropometrics

Height, weight, and body composition were assessed during V2. Height and weight were assessed using a stadiometer and scale (Health-o-meter Professional Patient Weighing Scale, Model 500 KL, Pelstar, Alsip, IL, USA) and body composition was assessed using bioelectrical impedance analysis (BIA; InBody 770, Biospace Co, Ltd. Seoul, Korea). For the BIA assessment, participants were asked to be at least 2 h fasted and well hydrated. Prior to testing, participants were asked to void their bladder and to remove shoes, socks, and all jewelry.

#### Maximum aerobic power test

During V3, participants performed a ramp protocol to volitional exhaustion on a cycle ergometer (Lode, Excalibur Sport, Groningen, The Netherlands). Prior to the MAP test, participants completed a warm-up consisting of 5 min of light cycling at an intensity of 50 watts (W) at a self-selected pace, 10 body weight squats, 10 body weight walking lunges, 10 dynamic walking hamstrings stretches, and 10 dynamic walking quadriceps stretches. Participants were then fitted with a heart rate monitor (chest strap and sensor; Polar H10, Polar Electro Oy, Kempele, Finland) just below the sternum at the xiphoid process to assess heart rate. The MAP test protocol required each participant to maintain a pedaling cadence of 70–80 revolutions per minute (RPM) at an initial workload of 100 W. The workload was increased by 1 W every 2 seconds (30 W/min) until the participant was unable to maintain a cadence above 70 RPM for 10 seconds despite verbal encouragement or reached volitional fatigue. Seat height was recorded for all participants and standardized during subsequent cycle assessments. PPO was determined as the highest power output achieved in watts by the participant at volitional exhaustion. Expired gases were analyzed using open-circuit spirometry (True One 2400^®^ Metabolic Measurement System, Parvo-Medics Inc., Sandy, UT) power output at the gas exchange threshold (GET). Participants were connected to the metabolic cart via a breathing tube connected to a measured and fitted facemask covering both the nose and mouth. GET was determined via computerized regression analysis of the slopes of CO_2_ uptake (VCO_2_) vs. O_2_ (VO2) uptake. Power at the GET was recorded.

#### High-intensity sprint exercise protocol

Prior to completion of the HISEP on a cycle ergometer (Lode, Excalibur Sport, Groningen, The Netherlands) participants underwent a standardized warmup identical to that performed prior to the MAP test. Resistance during the HISEP was set as a function of pedaling rate using a scaling factor based on the power output at a set cadence of 80 RPM being equal to 50% of the difference between the power output at GET and PPO assessed during the MAP test (Jeukendrup et al., 1996; Moon et al., 2023). The HISEP began with the participants completing a preparation phase on the cycle ergometer where they pedaled at 70–80 RPM for 1 min at a set resistance of 50 watts. During the last 5 seconds of the preparation phase, participants were instructed to begin pedaling as maximally as possible. Participants were blinded to their current RPMs and the elapsed time of the assessment and instructed to give maximal effort throughout the testing period and to not pace themselves. Strong verbal encouragement was provided throughout the duration of the assessment and was consistent for all participants. Upon completion of the HISEP, participants remained on the cycle ergometer and completed 3 min of unloaded cycling at a self-selected pace.

### Cognitive assessments

All cognitive assessments were carried out in a private quiet and dimly lit testing room, free of distractions at PRE, POST, and 60POST. The POST cognitive battery began no more than 5 min (including 3-min cool-down period) following the conclusion of the HISEP. Upon completion of the POST assessments, participants remained within the testing room until commencement of the 60POST assessments.

#### Dynavision D2 assessments

Psychomotor reaction time was assessed using the D2 visuomotor training device. The D2 consists of a 4 ft × 4 ft computer integrated board with 64 tactile light emitting targets arranged into five concentric rings. During a test, illuminated targets serve as a visual stimulus that require a physical hand strike to extinguish.

Mode A: The Mode A proactive reaction time task required participants to recognize and respond as fast as possible to random and sequential appearing stimulus across the Dynavision apparatus target field. Following a 5-second visual countdown on the screen in the center of the D2 board (t-scope), an initial stimulus presented on the D2 board in a random location. The stimulus remained illuminated until the button was struck by the participant, following which the stimulus appeared in another random location. The participant was instructed to successfully identify and strike as many stimuli as possible within 60 seconds with both hands. The number of hits and the average reaction time per hit (avgRT) were recorded for each test. The average of three discrete tests was used for each assessment at each time point.

Mode B: Similar to Mode A, the Mode B reactive reaction time task required participants to respond as fast as possible to random and sequential stimuli within the target field with the visual stimulus moving each time the target was successfully struck. However, during the Mode B assessment, the stimulus remained lit for only one second before automatically changing to another random location within the target field. In addition, the Mode B test included a cognitive stressor in the form of a five-digit number that participants were required to recite during the test. The 5-digit number was presented on the t-scope of the D2 apparatus 11 times during each 60 second trial and remained on the screen for 0.75 seconds. The number of hits, avgRT per hit, and number of misses were recorded for each test. The average of three discrete tests was used for each assessment at each time point.

To eliminate learning and training effects, participants completed ten Mode A assessments during V2 and eight additional Mode A assessments during V3 (Wells and Johnson, 2022). Additionally, three Mode B assessments were completed during V3 (Wells et al., 2014). Mode A and Mode B assessments required 8 min and were completed at PRE, POST, and 60POST.

#### Profile of mood states questionnaire

Mood state was assessed through the administration of the POMS paper questionnaire. The POMS consists of 58 words or phrases in a Likert format soliciting responses regarding how the participant feels at the time of completion (0–4; 0 = Not at all, 1 = A little, 2 = Moderately, 3 = Quite a bit, 4 = Extremely) and provides measures of tension, depression, anger, vigor, fatigue, and confusion. Total mood disturbance (TMD) was calculated by subtracting vigor from the sum of the 5 other negative mood states and adding 100 to avoid a negative result. Participants completed a POMS questionnaire at PRE, POST, and 60POST. The POMS questionnaire required 8 min to complete.

#### Automated Neuropsychological Assessment Metrics assessments

Cognitive performance was assessed using ANAM software (ANAM v.4.0; Vista Life Sciences, Parker, CO). The ANAM core battery was used for this study, which consisted of a concussion symptoms index (CSI) and eight cognitive subtests. The list of subtests, abbreviations, and measures/cognitive domains associated with the ANAM core battery are presented in Table 1. The CSI was used to assess the degree of psychological stress and consisted of 12 symptoms scored on a 7-point Likert type scale from 0 (absent) to 6 (severe). Symptoms included headache, nausea, balance problems/dizziness, fatigue, drowsiness, feeling like “in a fog,” difficulty concentrating, difficulty remembering, sensitivity to light and noise, blurred vision, and feeling slowed down. Previous literature has indicated that post concussion-like symptoms are not unique to mild head injury, with an increase in self-reported symptoms having been reported in healthy individuals who are experiencing high levels of perceived stress or mental fatigue (Balasundaram et al., 2017; Siddique et al., 2020).

**Table 1:** ANAM^TM^ Battery subtests and associated measures/cognitive domains.

**Subtest**	**Abbreviation**	**Measure/cognitive domain**
Concussion symptom inventory—frequency of endorsed symptoms	CSIfreq	Psychological stress (Wells et al., 2020)
Concussion symptom inventory—sum of severity ratings	CSIsum	Psychological stress (Wells et al., 2020)
Simple reaction time	SRT	Basic visuomotor processing speed (Vincent, 2015; Anders et al., 2021) attention (Vincent, 2015; Anders et al., 2021)
Code substitution-learning	CSL	Associative learning (Vincent, 2015; Anders et al., 2021; Venezia et al., 2023), visual scanning and perception (Vincent, 2015; Wells et al., 2020; Anders et al., 2021; Venezia et al., 2023), attention (Vincent, 2015; Wells et al., 2020; Anders et al., 2021; Venezia et al., 2023), information processing speed (Vincent, 2015; Wells et al., 2020; Anders et al., 2021; Venezia et al., 2023), and working memory (Wells et al., 2020)
Mathematical processing	MP	Computational skills (Vincent, 2015; Anders et al., 2021), working memory (Bue-Estes et al., 2008; Vincent, 2015; Anders et al., 2021), and concentration (Vincent, 2015; Anders et al., 2021)
Code substitution-delayed	CSD	Delayed visual recognition memory (Vincent, 2015; Anders et al., 2021), learning (Wells et al., 2020; Anders et al., 2021), and sustained attention (Wells et al., 2020)
Procedural reaction time	PRT	Basic visuomotor reaction time (Vincent, 2015; Anders et al., 2021), simple decision making (Vincent, 2015; Anders et al., 2021), and attention (Vincent, 2015; Anders et al., 2021)
Matching to sample	M2S	Visual-Spatial memory (Bue-Estes et al., 2008; Vincent, 2015), visual spatial processing (Vincent, 2015; Wells et al., 2020), and working memory (Vincent, 2015; Wells et al., 2020)
Simple reaction time-repeat	SRT2	Basic visuomotor processing speed (Vincent, 2015; Anders et al., 2021), attention (Vincent, 2015; Anders et al., 2021), and cognitive fatigue (Wells et al., 2020)
Go/no-go	GNG	Response inhibition (Vincent, 2015; Anders et al., 2021)

ANAM^TM^, Automated Neuropsychological Assessment Metrics.

Participants completed familiarization with the ANAM test battery during V2 and V3 to establish reliable testing and baseline cognitive scores, which is consistent with previous literature (Kaminski et al., 2009; Vincent et al., 2018). The test battery took approximately 30 min to complete with scoring and testing administered in accordance with the ANAM test manual. All ANAM subtests provided throughput scores (TP) that were used for analyses, except for the Go/No-Go test, and the Concussion Symptoms Inventory (CSI). Throughput (defined as LegacyThru in ANAM^TM^ software) represents the rate of correct responses per minute and is calculated using accuracy and speed variables, where speed is calculated by dividing 60,000 by the mean reaction time for all valid responses. Throughput is considered a measure of effectiveness or cognitive efficiency (Thorne, 2006). For the Go/No-Go test, D-prime scores were used, which represents the most comprehensive measure of accuracy on go and no-go trials by assessing the ability to detect and respond quickly to appropriate stimuli and inhibit inappropriate responses (Logan et al., 1997; Hinton et al., 2018). ANAM cognitive tests were completed at PRE, POST, and 60POST.

### Statistical analysis

A one-way repeated measures ANOVA was conducted to compare all dependent variables across time. In the event of a significant interaction, Bonferroni pairwise comparisons were used to assess changes in dependent variables across time. Prior to analyses, data was assessed for normality using the Shapiro Wilks test. All non-normally distributed data were log transformed and rechecked for normality. If data remained non-normally distributed, non-transformed data were used for analysis. If the assumption of sphericity was violated, a Greenhouse-Geiser correction was applied. Effects were further analyzed using partial eta-squared ($\eta_{\text{p}}^{2}$) and Hedges' *g* (*g*) effect sizes. Partial eta-squared were evaluated in accordance with Cohen (1988) at the following levels: small (0.01–0.058), medium (0.059–0.137), and large (>0.138) effects. Hedges *g* were interpreted using thresholds of < 0.2, 0.2 to < 0.6, 0.6 to < 1.2, 1.2 to < 2.0, and 2.0 to 4.0, which correspond to trivial, small, moderate, large, and very large ES, respectively. Since estimates for *g* may show positive bias with small sample sizes, a correction was applied to provide a more accurate estimate of effect size (Equation 1) (Wells et al., 2020).


(1) $$\begin{array}{l} g=\frac{{\bar{X}}_{2}-{\bar{X}}_{1}}{\sqrt{\frac{\left(n_{1}-1\right)S_{1}^{2}\;+\left(n_{2}-1\right)S_{2}^{2}\;}{\left(n_{1}-1\right)+\left(n_{2}-1\right)}}}\;\times \;1-\frac{3}{4n-9} \end{array}$$

Where n= number of observations for each time point/treatment and s= SD of the observations. All statistical analyses were completed using SPSS statistical software (v. 28.0.1.1). Significance for all analyses was accepted at an alpha level of *p* ≤ 0.05.

## Results

### High-intensity sprint exercise outcomes

HISEP Performance outcomes are provided in Table 2.

**Table 2:** HISEP variables.

**Variable**	**Mean** ±**(SD)**		
PP (W)	492	±	288
TPP (s)	4.82	±	5.17
WEP (kJ)	10.0	±	5.7
EP (W)	130	±	52
FI (%)	69	±	12

Values are shown as the mean ± standard deviation. PP, peak power; TPP, time to peak power; WEP, work above end power; EP, end power; FI, fatigue index; W, watts; s, seconds; and kJ, kilojoules.

### Psychomotor reaction time

**Figure 2:**
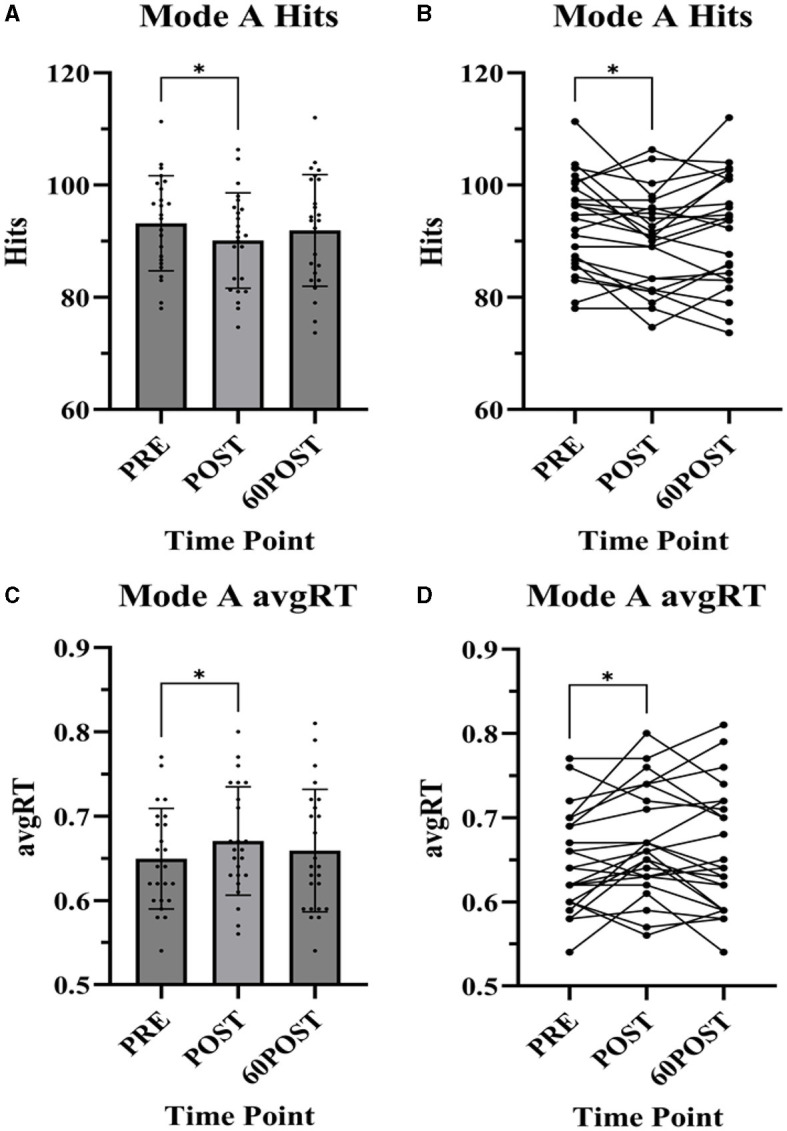
Dynavision D2 Mode A task assessments. Data presented as mean ± standard deviation. PRE, pre-exercise; POST, post-exercise; 60POST, 60 minutes post-exercise; avgRT, average reaction time; *, significantly different from PRE. **(A)** Mode A Hits, **(B)** Mode A Hits individual plots, **(C)** Mode A avgRT, and **(D)** Mode A avgRT individual plots.

Changes in Mode A variables are shown in Figure 2. Changes in Mode B variables are provided in Table 3.

**Table 3:** Dynavision D2 Mode B task assessments.

**Task**	**PRE**	**POST**	**60POST**
Dynavision Mode B Hits	77.40 ± 11.34	76.54 ± 11.73	77.61 ± 10.70
Dynavision Mode B avgRT (sec)	0.66 ± 0.04	0.67 ± 0.05	0.66 ± 0.04
Dynavision Mode B misses	8.65 ± 4.63	9.15 ± 4.59	8.56 ± 4.33

Data presented as mean ± standard deviation. PRE, pre-exercise; POST, post-exercise; 60POST, 60 minutes post-exercise.

#### Mode A

A significant time effect was observed for the number of hits [*F*_(2,46)_ = 4.671, *p* = 0.014, $\eta_{\text{p}}^{2}$ = 0.758] and avgRT [*F*_(2,46)_ = 4.142, *p* = 0.022, $\eta_{\text{p}}^{2}$ = 0.153] in the Mode A test. The number of hits was significantly lower (*p* = 0.029, g = −0.356) and avgRT was significantly slower (*p* = 0.036, g = 0.330) at POST compared to PRE. No significant differences were noted between 60POST and PRE or 60POST and POST for either variable (*p's* > 0.05).

#### Mode B

No significant time effects were noted for hits [*F*_(2,46)_ = 0.689, *p* = 0.689, $\eta_{\text{p}}^{2}$ = 0.159], misses [*F*_(2,46)_ = 0.856, *p* = 0.431, $\eta_{\text{p}}^{2}$ = 0.036], or avgRT [*F*_(2,46)_ = 1.149, *p* = 0.326, $\eta_{\text{p}}^{2}$ = 0.240] in the Mode B test.

### Mood states (POMS)

Change in Profile of Mood States are provided in Table 4. Significant time effects were observed for tension [*F*_(2,46)_ = 4.182, *p* = 0.021, $\eta_{\text{p}}^{2}$ = 0.154] and fatigue [*F*_(2,46)_ = 10.310, *p* < 0.001, $\eta_{\text{p}}^{2}$ = 0.309]. Tension scores were significantly lower at 60POST when compared to PRE (*p* = 0.012, g = −0.310), while fatigue scores were significantly higher at POST when compared to PRE (*p* = 0.005, g = 0.586). No other significant differences were noted between time points for tension or fatigue (*p's* > 0.05). Additionally, no significant effects for time were observed for depression, anger, vigor, confusion or TMD.

**Table 4:** Changes in mood states as assessed via the Profile of Mood States questionnaire.

**Mood state**	**PRE**	**POST**	**60POST**
Tension	38.75 ± 6.08	37.88 ± 8.45	36.71 ± 6.84^*^
Depression	38.04 ± 2.44	37.75 ± 2.56	37.46 ± 1.50
Anger	38.83 ± 5.41	38.71 ± 6.58	39.08 ± 6.23
Vigor	46.79 ± 10.88	46.67 ± 12.73	45.38 ± 12.60
Fatigue	38.54 ± 7.14	43.67 ± 9.86^*^	37.21 ± 4.64
Confusion	34.92 ± 4.79	35.21 ± 6.49	34.54 ± 5.83
TMD	242.29 ± 26.17	246.54 ± 30.18	239.63 ± 20.79

Data presented as mean ± standard deviation. PRE, pre-exercise; POST, post-exercise; 60POST, 60 minutes post-exercise; ^*^, significantly different from PRE.

### Automated Neuropsychological Assessment Metrics

**Figure 3:**
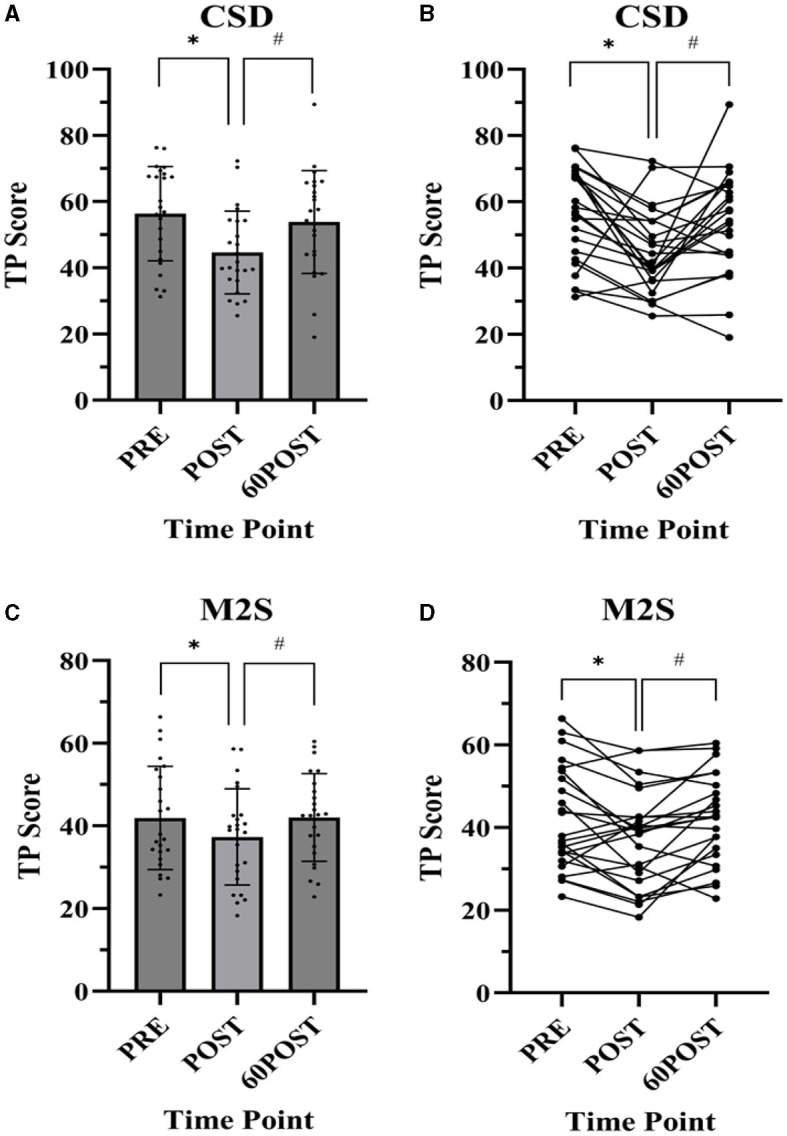
Changes in Automated Neuropsychological Assessment Metrics (ANAM) Data presented as mean ± standard deviation. PRE, pre-exercise; POST, post-exercise; 60POST, 60 minutes post-exercise; M2S, matching to sample; CSD, Code substitution-delayed; *, significantly different from PRE; #, significantly different from POST; **(A)** M2S, **(B)** M2S individual plots, **(C)** CSD, **(D)** CSD individual plots.

Changes in CSI and ANAM variables are provided in Table 5. Relevant significant changes in ANAM variables are shown in Figure 3.

**Table 5:** Changes in Automated Neuropsychological Assessment Metrics (ANAM) data presented as mean ± standard deviation.

**ANAM task**	**PRE**	**POST**	**60POST**
SRT1 (TP)	217.38 ± 25.34	205.70 ± 37.66	210.07 ± 24.49
CSL (TP)	62.98 ± 11.63	58.43 ± 11.56	62.50 ± 10.05
PRT (TP)	99.90 ± 12.58	101.60 ± 16.29	104.29 ± 11.84
MP (TP)	28.06 ± 9.81	26.89 ± 9.07	31.05 ± 8.63^#^
SRT2 (TP)	205.83 ± 29.83	205.72 ± 24.91	208.29 ± 23.90
GNG (D-Prime)	4.26 ± 1.69	3.63 ± 1.38	3.63 ± 1.41
CSIfreq	14.81 ± 19.57	12.96 ± 18.14	10.18 ± 18.51
CSIsev	2.79 ± 4.75	2.21 ± 4.06	1.83 ± 4.40^*^

PRE, pre-exercise; POST, post-exercise; 60POST, 60 minutes post-exercise; ^*^, significantly different from PRE; #, significantly different from POST; SRT1, simple reaction time; CSL, code substitution learning; PRT, procedural reaction time; MP, mathematical processing; SRT2, simple reaction time repeat; GNG, go/no-go; CSIfreq, frequency of concussion-like symptoms; CSIsev, severity of concussion-like symptoms.

#### Concussion Symptoms Inventory

There was a significant time effect for the sum of severity ratings for CSI symptoms (CSIsum) [*F*_(2,46)_ = 3.636, *p* = 0.034, $\eta_{\text{p}}^{2}$ = 0.137]. CSIsum was significantly lower at 60POST compared to PRE (*p* = 0.037, g = −0.206). No significant differences in CSIsum were observed between PRE and POST or POST and 60POST (*p* > 0.05). No significant change in the number of endorsed CSI symptoms (CSIfreq) was observed in response to the HISEP (*p* > 0.05).

#### ANAM cognitive tasks (core battery)

Significant time effects were observed for CSD [*F*_(2,46)_ = 9.103, *p* < 0.001, $\eta_{\text{p}}^{2}$ = 0.284], M2S [*F*_(2,46)_ = 4.687, *p* = 0.014, $\eta_{\text{p}}^{2}$ = 0.169], and MP [*F*_(2,46)_ = 6.965, *p* = 0.002, $\eta_{\text{p}}^{2}$ = 0.232] tasks. In CSD and M2S tasks, TP was significantly lower at POST compared to PRE (CSD: *p* = 0.001, g = −0.876; M2S: *p* = 0.025, g = −0.376) and significantly greater at 60POST compared to POST (CSD: *p* = 0.016, g = 0.643; M2S: *p* = 0.007, g = 0.416), with no differences between PRE and 60POST (*p's* > 0.05). In the MP task, TP was significantly greater at 60POST compared to both PRE (*p* = 0.019, g = 0.318) and POST (*p* = 0.005, g = 0.462), but was not different between POST and PRE (*p* > 0.05). No significant time effects were noted for TP in CSL, SRT1, SRT2, or PRT tasks, or for D-prime scores in the GNG task (*p's* > 0.50).

## Discussion

The purpose of this investigation was to examine the impact of a HISEP in eliciting acute cognitive dysfunction on indices of psychomotor reaction time, mood, and cognition. Deficits in psychomotor reaction time (Mode A hits and avgRT) and TP scores in CSD and M2S ANAM tasks were observed immediately following the HISEP, although these deficits were no longer apparent at 60P, indicating a return to pre-HISEP scores. These deficits coincided with a simultaneous increase in reported fatigue at POST compared to PRE. In contrast, TP scores in CSL, SRT, PRT and MP tasks, and D-Prime in the GNG task did not appear to be affected by the HISEP, while TP in the MP task and feelings of tension were improved following the HISEP. Collectively, these results indicate that the HISEP was a fatiguing stimulus which successfully evoked relatively transient (< 60 min) post-exercise deficits in psychomotor reaction time performance (Mode A), memory recall (CSD), and visual spatial memory recall (M2S), but not MP ability, GNG, SRT, or PRT. The HISEP may therefore have utility as an acute intervention aimed at eliciting acute cognitive dysfunction in psychomotor and memory recall tasks.

Although the effects of exercise on cognitive performance have been extensively investigated (Lambourne and Tomporowski, 2010; McMorris and Hale, 2012; McMorris et al., 2015; Sudo et al., 2022), to our knowledge, no study has used an exercise protocol that closely matches the HISEP or included such a robust variety of cognitive assessments following said exercise protocol. As such, the ability to draw conclusions from direct comparisons is limited. Changes in cognitive performance have, however, been examined in response to several staged high-intensity exercise protocols. Coco et al. (2020) examined the effect of a multistage discontinuous incremental cycling protocol on simple reaction time among 15 young adults using a computer based SRT task. Exercise was performed on a mechanically braked cycle ergometer at a pedaling cadence of 60 rpm with load increases of 30 W every 3 min until volitional exhaustion. They reported significantly slower simple reaction times immediately post-exercise, which is in contrast with our findings using an equivalent SRT task (ANAM SRT). In another study, Mekari et al. (2015) examined changes in psychomotor reaction time among 16 young adults following a high-intensity stepping protocol. Following an initial cadence of 100 steps per minute (spm), stepping rate was increased by 10 spm every 60s until participants reached an RPE of 9 (scale of 1–10). A Dynavision task similar to our Mode B task was utilized, albeit with an alternative cognitive stressor (solving a simple mathematical equation vs. reciting a 5-digit number) and a visual field narrowed to include only the inner three rings of the Dynavision board. Consistent with our Mode B results, no differences in reaction time following the stepping protocol were observed. The implications of differences in Dynavision task parameters are therefore unclear. The current examination is one of a small number of studies to utilize the ANAM when assessing cognition following acute high-intensity exercise. Previously, Bue-Estes et al. (2008), examined the effects of short-term discontinuous maximal treadmill running to exhaustion on ANAM SRT, CSL, MP, CSD, and M2S task throughput scores in 18 young adult women. Consistent with the current investigation, no significant effects of high-intensity exercise were observed on the SRT and CSL tasks post-exercise. However, in contrast to our findings, a decrease in MP was observed at POST, while CSD and M2S tasks were unaffected. Interestingly, both investigations showed significantly improved MP following a recovery period when compared to POST. Disparities in findings between investigations may be attributed to several key moderating variables identified by previous meta-analysis and reviews (Lambourne and Tomporowski, 2010; Browne et al., 2017; Sudo et al., 2022), including the cognitive task utilized (Lambourne and Tomporowski, 2010; Sudo et al., 2022), exercise modality (Lambourne and Tomporowski, 2010; Browne et al., 2017; Sudo et al., 2022), exercise intensity (Lambourne and Tomporowski, 2010), time elapsed between the exercise stressor and the cognitive task (Lambourne and Tomporowski, 2010; Sudo et al., 2022), and the fitness level of the population examined (Browne et al., 2017; Sudo et al., 2022). Therefore, differences in the observed cognitive outcomes following acute exercise between the current investigation and others may be explained in part by the disparity in cognitive tasks and the exercise stimuli utilized. Additionally, inconsistencies in the duration between the end of the exercise stimuli and the completion of the cognitive task may further distort comparisons. For example, due to the order of post-exercise assessments of the current investigation, ANAM subtests at POST were administered ~25 min following the exercise bout, which is in notably different than the 3 min reported by Bue-Estes et al. (2008). Finally, participants who have higher baseline fitness levels have been shown to be more resilient to cognitive deficits compared to their less fit counterparts following exercise in a variety of cognitive tasks (Brisswalter et al., 1997; Sudo et al., 2022). Considering that the population of the current investigation and those done previously were not matched for fitness levels, this may further explain our contradictory findings. Thus, cognitive outcomes are seemingly highly specific to the implemented experimental design of an intervention (Lambourne and Tomporowski, 2010; Sudo et al., 2022) and comparisons between investigations may need to be considered with caution unless designs are strictly matched for modifying parameters.

Harvey (2019) proposed that cognitive domains do not function independently of one another and that many higher order functions rely on lower order operations for optimal performance. However, the exact magnitude of synergy in which these domains function has not been fully elucidated (Harvey, 2019). Additionally, the definitional descriptions of each of these domains can be ambiguous, which has led to inconsistencies in the classification of cognitive tasks and their corresponding domains within the literature. For example, the CSL task has been separately categorized as an assessment of executive function (Wells et al., 2020) and attention (Venezia et al., 2023). Considering this, the approach taken by Sudo et al. (2022) may be the most appropriate when evaluating cognitive outcomes following exercise. They propose a simplified categorization of cognitive tasks into four categories consisting of attentional, executive function, memory, and psychomotor tasks. Thus, our cognitive tasks were dichotomized using the four-category approach in accordance with their definitional descriptions, previous literature, and the outcomes observed within the current investigation (Table 6). Nevertheless, stratification within these four categories is not entirely mutually exclusive (i.e., psychomotor tasks commonly assess attention simultaneously) (Vincent, 2015; Anders et al., 2021). Therefore, within this categorization, outcomes may be best interpreted as a representation of the effects of the HISEP on multiple domains working simultaneously to complete the cognitive task rather than its effects on an individual cognitive domain.

**Table 6:** Categorization of cognitive tasks.

	**Attentional**	**Executive function**	**Memory**	**Psychomotor**
Dynavision Mode A	•			•
Dynavision Mode B	•			•
ANAM SRT	•			•
ANAM CSL	•	•		
ANAM MP		•		
ANAM CSD	•		•	
ANAM PRT	•			•
ANAM M2S		•	•	
ANAM SRT2	•			•
ANAM GNG		•		

Symbols denote which category (4) each cognitive task within the current investigation assessed. ANAM, Automated Neuropsychological Assessment Metrics; SRT, simple reaction time; CSL, code substitution-learning; MP, mathematical processing; CSD, code substitution-delayed; PRT, procedural reaction time; M2S, matching to sample; GNG, Go/No-Go.

Sudo et al. (2022) reported an absence of a clear association between cognitive task and cognitive performance following high-intensity exercise in their narrative review. Nevertheless, impairments in psychomotor tasks were observed more often than in attentional tasks following high-intensity exercise (Sudo et al., 2022). Consistent with this, we observed no significant changes in many of our attentional tasks (Mode B, SRT, PRT, or SRT2). However, there was a significant reduction in our Mode A task at POST. These seemingly contradictory findings may instead indicate that the relatively large motor component of the Mode A task compared to the SRT and PRT tasks (arm swing vs. button click) requires a proportionally greater psychomotor vs. attentional demand. In consideration of this, similar outcomes were expected in the Mode B assessment, yet we observed no significant differences in Mode B performance from pre- to post-HISEP. Importantly, both Mode A and B tasks require sustained attention to remain vigilant for the randomly illuminating stimuli (lights). However, the Mode B tasks also requires selective attention in order to correctly recite the t-scope 5-digit code while performing the psychomotor component of the task. Therefore, success during the Mode B task may be more influenced by attentional capacity, which may explain why even though seemingly analogous to the Mode A task, Mode B outcomes were unaffected by the HISEP.

Although associations are not clear (Sudo et al., 2022), reduced executive function task performance has been shown immediately following high-intensity exercise (Labelle et al., 2013). Dietrich and Audiffren (2011)'s hypofrontality theory partially explains this phenomenon by proposing that high-intensity exercise shifts the finite allocation of metabolic resources in the brain toward the motor task itself and away from explicit processes, such as executive function and emotion, that rely on prefrontal regions such as executive function (Dietrich and Audiffren, 2011; Anders et al., 2021; Sudo et al., 2022). Therefore, we expected to see post-exercise reductions in at least one of our primary executive function tasks (CSL, MP, and GNG). However, the cerebral metabolic environment has been shown to return to baseline rapidly following exercise (Dietrich and Audiffren, 2011; Curtelin et al., 2018; Sudo et al., 2022), which indicates that the elapsed time between the HISEP and commencement of the ANAM assessment in the current investigation (~25 min) likely permitted cerebral recovery. Therefore, the current investigation likely did not truly capture the effects of the HISEP on executive function immediately following high-intensity exercise.

Elevated concentrations of dopamine, noradrenaline, and brain derived neurotrophic factors are required for potentiation and long term memory consolidation (McMorris, 2021). Because of this, previous studies have proposed that the facilitation of memory tasks commonly observed following exercise may in part be due to the elevated concentrations of these markers elicited by high-intensity exercise (Winter et al., 2007; Skriver et al., 2014; Hwang et al., 2016; Sudo et al., 2022). Notwithstanding, these responses are poorly characterized immediately following acute high-intensity exercise and a conclusion regarding their effects has yet to be fully elucidated (Sudo et al., 2022). Within the current investigation, we observed reductions in our memory tasks (CSD and M2S) at POST. Additionally, no significant changes in our CSL task were observed at POST. These findings are consistent with those observed by Anders et al. (2021) and indicate that high-intensity exercise may diminish short term memory recall independently of the learning process. While the exact mechanisms responsible for acute disruption of cognition following high-intensity exercise are not fully understood, it has previously been proposed that a myriad of metabolic, hemodynamic, and hormonal factors may be contributing (Sudo et al., 2022). Coco et al. (2020) propose that elevated cerebral concentrations of lactate may induce diminished cerebral function in select areas of the brain. Elevated cerebral lactate concentrations are also posited to provide an alternative energy source for neuronal tissue and has been shown to facilitate cognition in attentional tasks (Herold et al., 2022). Hyperventilation during high-intensity exercise has been shown to induce cerebral vasoconstriction (Smith and Ainslie, 2017). Additionally, although contested (Ando et al., 2011, 2013), cerebral hypoxia during high-intensity exercise has also been shown to elicit cognitive dysfunction (Labelle et al., 2013). However, considering cerebral blood flow and oxygenation has been shown to recover rapidly following exercise (Curtelin et al., 2018), these hemodynamic factors likely do not contribute to post-exercise cognitive dysfunction directly but instead may induce dysfunction in combination with other phenomena (Sudo et al., 2022). Concentrations of noradrenaline, serotonin, and dopamine are thought to follow a sharp inverted-U relationship with cognitive function of the prefrontal cortex (Arnsten, 2011; Cools and Arnsten, 2022). Consequentially, elevated concentrations of these neuromodulators induced by high-intensity exercise have also been posited to be responsible for post-exercise cognitive dysfunction (Sudo et al., 2022) and may help to explain the reductions in psychomotor and memory tasks observed within the current investigation. Taken together, these conflicting findings further demonstrate the specificity and complexity of the relationship between high-intensity exercise and cognition and may help to explain why contradictory results were observed within the current investigation between seemingly analogous tasks (e.g., Dynavision Mode A and Mode B). Unfortunately, the current investigation did not collect measures of the cerebral environment and therefore we cannot characterize what perturbations are directly responsible for observed outcomes in our cognitive tasks.

Previously, researchers examining the effects of exercise on cognition have utilized a plethora of different treadmill and cycle-based protocols yielding mixed cognitive outcomes (Lambourne and Tomporowski, 2010; Sudo et al., 2022). However, high-intensity cycling appears to elicit cognitive dysfunction more often than cognitive enhancement (Sudo et al., 2022), while high-intensity running is more consistently faciliatory (Sudo et al., 2022). Furthermore, Mekari and colleagues (Labelle et al., 2013) demonstrated that increasing the intensity of a cycling exercise stimulus from 40% to 80% PPO intensifies the cognitive dysfunction elicited, indicating a maximal effort stimulus may be optimal for evoking the greatest cognitive dysfunction. Although the minimal differences in cognitive outcomes between treadmill running and cycling may not solely warrant the exclusive use of cycling in future investigations, treadmill running is also less kinematically stable (Sudo et al., 2022) and can be uncomfortable for individuals concerned with falling (Miller et al., 2019). Therefore, in light of the findings of the current investigation, and considering cerebral perturbations are greatest following exercise that is completed at the highest possible intensity (Dietrich and Audiffren, 2011), the stability and continually maximal effort of the HISEP may warrant its implementation in investigations seeking to elicit cognitive dysfunction for the purpose of assessing interventions designed to mitigate these effects.

### Limitations

The study sample of the current investigation consisted of a sample of recreationally fit men and women. As such, the findings of this study may not be generalizable to individuals of different fitness levels. Differences in the fitness levels and exercise frequency among participants in our sample may also have varied among participants despite meeting ACSM criteria for recreationally active individuals. Although consistent verbal encouragement was provided during the HISEP for all participants, heterogeneity even among recreationally fit individuals may have influenced execution of the HISEP and subsequent cognitive outcomes. We did not collect data on training history and are therefore unable to assess any potential influence of prior training on study outcomes; however, there were no indications that a maximal effort was not provided from any of the participants. Although it is generally recommended to include a familiarization trial for maximal assessments, previous data from a sub group of participants within the current study's sample showed no significant differences in performances variables between a 1^st^ and 2^nd^ HISEP bouts (Dufner et al., 2023). Furthermore, subsequent unpublished reliability analysis of peak power, work above end power, end power, and fatigue index showed good to excellent reliability (ICC_2,1_ = 0.860–0.990) indicating a familiarization trial may not necessarily be required to facilitate a truly maximal effort during the HISEP. Additionally, differences in participant fitness levels may have influenced the degree to which cognitive dysfunction was elicited, as those with higher levels of fitness may be more resilient to the cognitive stress imposed by the HISEP (Brisswalter et al., 1997; Basso and Suzuki, 2017; Sudo et al., 2022; Dufner et al., 2023). However, analysis of correlations between MAP and changes in cognition from PRE indicated no significant associations between fitness level and cognitive responses to the HISEP (data not shown). Although no control group was included in the current investigation, participants were rigorously familiarized with all cognitive assessments including 2 complete familiarizations with the ANAM and 13 total familiarizations with the Dynavision psychomotor tasks. Previous investigation indicate these familiarizations are sufficient to mitigate the possibility of learning effects (Kaminski et al., 2009; Anders et al., 2021; Wells and Johnson, 2022). Moreover, we observed deleterious effects of the HISEP on several ANAM tasks. Alternatively, it has been previously documented that repetitive cognitive testing may induce cognitive fatigue that would not be attributable to the HISEP. However, a ≈10 min break was provided between the ANAM batteries administered at POST and 60POST, which has been shown to be long enough in duration to elicit cognitive recovery, even in the presence of continually decreasing motivation (Möckel et al., 2015). Furthermore, CSIfreq was unchanged from PRE at POST and 60POST, while CSIsum was lower at 60POST compared to PRE, indicating that cognitive stress was lower at 60P. Therefore, we do not expect that repeating the cognitive battery contributed to additional cognitive stress beyond that elicited by the HISEP. Nevertheless, including a control group may have helped to contextualize cognitive perturbations to the HISEP. Lastly, the current investigation did not have the power to assess any order effects. Therefore, although the order of the cognitive assessments may have affected the degree in which they were perturbed, we were unable to directly assess this relationship. Accordingly, findings of the current investigation may be best compared to cognitive batteries of similar length and order. To strengthen comparisons between investigations, future studies may wish to standardize the time elapsed between the exercise stressor and the cognitive tasks utilized or replicate the cognitive battery of a previous study.

## Conclusions

In conclusion, the HISEP is a feasible and time-effective fatiguing exercise stimulus capable of eliciting acute cognitive dysfunction in psychomotor (Mode A) and memory CSD & M2S) task performance in a recreationally active population. While many previous studies have examined cognition following high-intensity exercise (Bue-Estes et al., 2008; McMorris et al., 2009; McMorris and Hale, 2012; Labelle et al., 2013), this is the first to our knowledge to examine the impact of utilizing the HISEP as a stressor specifically meant to elicit cognitive dysfunction in addition to providing commentary on the transiency of the cognitive task effects. The observed effects of the HISEP on memory tasks within the current investigation may warrant its future use as a stimulus designed to elicit cognitive dysfunction in this domain. Finally, given that cognitive outcomes following exercise appear to be influenced by a variety of moderators (Lambourne and Tomporowski, 2010; Browne et al., 2017; Sudo et al., 2022), future studies should consider controlling for these variables when designing intervention studies to minimize the variability associated with cognitive responses to high-intensity exercise protocols.

## Data availability statement

The raw data supporting the conclusions of this article will be made available by the authors, without undue reservation.

## Ethics statement

The studies involving humans were approved by the University of Central Florida Institution Review Board. The studies were conducted in accordance with the local legislation and institutional requirements. The participants provided their written informed consent to participate in this study.

## Author contributions

TD: Conceptualization, Data curation, Formal analysis, Investigation, Methodology, Resources, Software, Validation, Visualization, Writing – original draft, Writing – review & editing. JM: Conceptualization, Data curation, Investigation, Methodology, Resources, Software, Validation, Writing – original draft, Writing – review & editing. AW: Conceptualization, Data curation, Formal analysis, Funding acquisition, Investigation, Methodology, Project administration, Resources, Software, Supervision, Validation, Visualization, Writing – original draft, Writing – review & editing.
